# Correction: Demographic-environmental effect on dengue outbreaks in 11 countries

**DOI:** 10.1371/journal.pone.0342211

**Published:** 2026-02-04

**Authors:** Anamul Haque Sajib, Sabina Akter, Goutam Saha, Zakir Hossain

With this Correction, the authors provide a revised Fig 1 to address concerns that the low resolution of the originally published Fig 1 limited its utility. No changes beyond improved image quality have been made to the content of the revised Fig 1.

**Fig 1:**
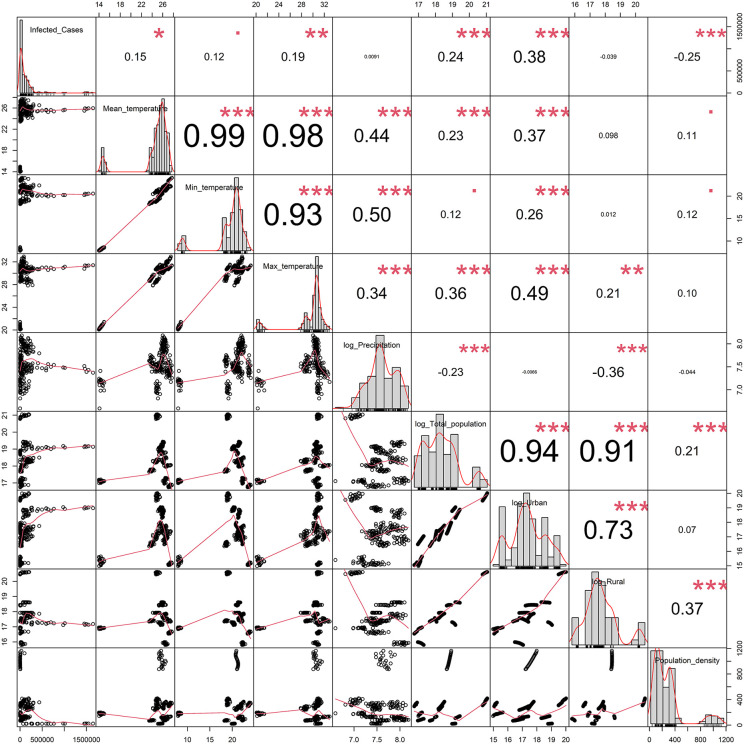
Pairwise correlation plot. (Note: p < 0.1*, p < 0.05**, p < 0.01**).

## References

[1] Sajib AH, Akter S, Saha G, Hossain Z. Demographic-environmental effect on dengue outbreaks in 11 countries. PLoS One. 2024;19(9):e0305854. doi: 10.1371/journal.pone.0305854 39259718 PMC11389931

