# Supplementary material for: Demographic-environmental effect on dengue outbreaks in 11 countries
Source: PLoS One. 2024 Sep 11;19(9):e0305854. doi: 10.1371/journal.pone.0305854 (PMC11389931; doi:10.1371/journal.pone.0305854)
Supplement: S1 Appendix — (DOCX) [file pone.0305854.s001.docx]

Appendix

Ministry of Health:

Bangladesh: <https://old.dghs.gov.bd/index.php/bd/>

Nepal: <https://mohp.gov.np/en>

Sri Lanka: <https://www.dengue.health.gov.lk/>

India: <https://ncvbdc.mohfw.gov.in/index.php>

Indonesia: <https://www.kemkes.go.id/eng/home>

Malaysia: <https://iku.moh.gov.my/>

Philippines: <https://doh.gov.ph/>

Thailand: <https://ddc.moph.go.th/viralpneumonia/eng/index.php>

Myanmar: <https://moh.nugmyanmar.org/>

Vietnam: <https://moh.gov.vn/web/ministry-of-health>
